# Peer specialists deliver cognitive behavioral social skills training compared to social skills training and treatment as usual to veterans with serious mental illness: study protocol for a randomized controlled trial

**DOI:** 10.1186/s13063-022-06376-9

**Published:** 2022-05-24

**Authors:** Chantele Mitchell-Miland, Sharon McCarthy, Matthew Chinman

**Affiliations:** 1 VA Pittsburgh Healthcare System University Drive Division, CHERP/MIRECC, Pittsburgh, PA USA; 2 University of Pittsburgh School of Medicine, 200 Meyran Ave., Suite 300, Pittsburgh, PA 15213 USA; 3 VA Pittsburgh Healthcare System University Drive Division, Health Science, Pittsburgh, PA USA; 4 RAND Corporation, Santa Monica, CA USA

## Abstract

**Background:**

Serious mental illness (SMI) affects 4.6% of the American population. While treatments are available, adherence to specific regimens is often suboptimal. Multiple organizations, such as the Substance Abuse and Mental Health Services Administration (SAMHSA), have called for more options that improve accessibility and engagement to treatment among individuals with SMI. This study protocol answers such calls by testing the effectiveness of peer specialists—individuals with SMI trained to use their experience to help others with SMI—in delivering social skills training (SST) and cognitive behavioral social skills training (CBSST), evidence-based treatments effective at engaging individuals with SMI to make behavioral and cognitive changes. Peer specialists have been shown to be adept at engaging those with SMI in treatment; however, their ability to deliver these structured treatments is unknown.

**Methods:**

This study is a randomized, hybrid 1, research assistant-blinded, superiority trial. A total of 252 veterans with SMI will be recruited and randomized to one of three arms: CBSST-Peer vs. SST-Peer vs. treatment as usual. Participants randomized to CBSST-Peer or SST-Peer will participate in a 20-week group-based intervention that meets weekly for a 60-min class. All participants will complete 4 study assessments at baseline, 10 weeks, 20 weeks, and 32 weeks. A multidimensional battery of functional outcomes will be used with the Independent Living Skills Survey (ILSS) as the primary outcome measure. Post-study completion, veterans who participated in the CBSST-Peer or SST-Peer arms will randomly be invited to participate in focus groups, and peer specialists will complete interviews to further assess the effectiveness of each intervention.

**Discussion:**

Improving care and outcomes for individuals with SMI is a national priority. To improve care, it is imperative to think about new ways to improve engagement and accessibility to care. This study provides an innovative solution to this problem by evaluating how two different types of treatment, delivered by peer specialists, compare to usual care. The results of the study will allow for the expansion of treatment options that improve access and engagement among veterans with SMI.

**Supplementary Information:**

The online version contains supplementary material available at 10.1186/s13063-022-06376-9.

## Background

Serious mental illness (SMI) encompasses many mental, behavioral, or emotional disorders that result in serious functional impairment and affects 11.4 million (4.6%) US adults aged 18 or older [1]. Treating SMI is often complicated. Medications can be effective in treating two hallmark symptoms—hallucinations and delusions; however, when solely used to manage illness, rates of non-adherence increase over time [2]. Those with SMI often have difficulty accessing care and often drop out. Due to the complicated nature of treatment and barriers to care, the Interdepartmental Serious Mental Illness Coordinating Committee, led by the Substance Abuse and Mental Health Services Administration (SAMHSA), called for enhanced efforts across five areas to improve care for those with SMI [3]. The second and fifth foci aim at improving access and engagement in care. Recommendations for better access to and engagement in care include the use of peer supports (i.e., individuals with SMI helping each other) and cognitive therapies (improving errors in thinking) and a movement towards understanding what is most effective. More research is needed to understand how best to use peer support to improve access to and engagement in care. This study offers an opportunity to fill the gaps in knowledge around peer support and effective behavioral and cognitive treatments.

Peer specialists—a particular type of peer support—are individuals in recovery from SMI who receive specialized training and use their lived experience to support others with SMI [4]. Peer specialists often collaborate with professionally credentialed mental health care providers to extend care beyond more traditional settings [5]. While peer specialists have been shown to contribute to improved care access and engagement among those with SMI, they are not credentialed to provide a full range of treatment. For this reason, research has focused on how to maximize the services they can provide. Chinman et al.’s [4] literature review of 20 studies showed that peer support services are especially effective when they use structured goal-setting curricula. One particular curriculum, cognitive behavioral social skills training (CBSST), provides an important opportunity to test peer specialists’ effectiveness in delivering a structured curriculum for patients with SMI.

CBSST [6] was developed by combining techniques derived from cognitive behavioral therapy (CBT) and social skills training (SST), including thought challenging, role-playing of communication skills, and problem-solving training, into a unique intervention that enhances daily role functioning. CBSST is a recovery-oriented psychosocial rehabilitation intervention that helps patients with SMI set goals, corrects errors in thinking, and builds communication skills to improve social functioning [7]. CBSST studies have demonstrated improved functioning, negative symptoms, and defeatist attitudes among individuals with schizophrenia and psychosis [7–13]. In these studies, CBSST was delivered by masters- or doctoral-level therapists, which limits how widely the intervention can be delivered.

Testing CBSST delivery by peer specialists provides several benefits. If effective, more providers will be able to deliver the intervention in a clinical setting, which meets SAMHSA’s third focus area. Additionally, peer specialists have the unique opportunity to use their lived experience while delivering the intervention which can enhance engagement among participants. Furthermore, this structured interface with SMI participants provides peer specialists with an ideal format to assist participants with achieving and maintaining recovery while improving functioning.

A recent pilot study using peer specialists to deliver CBSST showed improvement in outcomes among participants with SMI (*n* = 12) and demonstrated the ability of the peer specialists to deliver CBSST with high fidelity [14]. This study is the next step to examining the effectiveness of CBSST among individuals with SMI while also assessing implementation considerations in typical clinical settings. This study tests the effectiveness of peer specialists delivering CBSST (called CBSST-Peer) compared with SST-Peer and usual care among a population of veterans with SMI. Social skills training (SST) is another curriculum that targets some of the same social skills as CBSST but does not target errors in thinking (the “cognitive” in CBSST). Two meta-analyses of 49 SST clinical trials of patients with schizophrenia show that SST can improve basic conversation skills and community functioning [15, 16]. The following specific aims were developed based on the results of the pilot study.

### Study aims

The following are the study aims:

1. Compare the impact of CBSST-Peer on outcomes in veterans with SMI to veterans receiving SST-Peer and treatment as usual (TAU)
2. Use focus groups with study participants and interviews with peer specialists and other staff to assess perceptions of SST- and CBSST-Peer and identify the potential barriers and facilitators to future implementation

To date, no study has tested peer-delivered SST or CBSST or compared the two in a rigorous trial. Should this study show that either of these manualized treatments delivered by peer specialists are effective, it could increase the availability of evidence-based services veterans could receive.

## Methods

### Study design

This is a randomized, hybrid 1, research assistant (RA)-blinded, superiority trial involving 252 veterans with SMI from one Mid-Atlantic VA Medical Center comparing three arms: CBSST-Peer vs. SST-Peer vs. TAU. Hybrid 1 trials test the effectiveness of an intervention and collect implementation barriers and facilitators that could inform its future adoption [17].

**Fig. 1:**
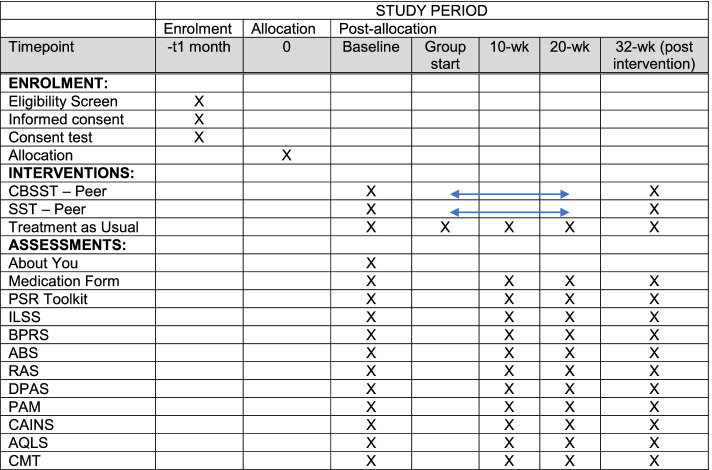
SPIRIT figure

After participants are randomized, they will be followed for 32 weeks—baseline, 10-week, 20-week, and 3-month post-intervention assessments (see Fig. 1). All research activities have been reviewed and approved by the Veterans Administration Central Institutional Review Board (IRB) and reviewed by local IRBs and Research & Development Committees. Participants will provide written informed consent at the first study visit. RAs will inform the participants about the reasons the research is being done, the study procedures, and the risks and benefits. Participants are encouraged to ask questions and must demonstrate their understanding of the study by passing a brief quiz prior to signing the consent. As part of the consent, participants are asked if they are willing to be contacted later about participation in a focus group. Significant study changes will be communicated to the participants by reconsent if necessary.

### Recruitment

Recruitment for this study takes place via a combination of IRB-approved advertisements placed throughout the VA Medical Center, provider referrals, and review of VA medical records via the VA’s Corporate Data Warehouse (CDW) using ICD10 codes of SMI diagnosis of interest to assess eligibility.

### Participant eligibility

The following inclusion criteria, selected to increase generalizability, will be used: (1) age 18 and older; (2) primary diagnosis of SMI documented in the medical record (schizophrenia, schizoaffective disorder, bipolar disorder with psychotic features, major depression with psychotic features, delusional disorder, schizophreniform disorder, another specified schizophrenia spectrum, and other psychotic disorders); and (3) fluency in English. The exclusion criteria are as follows: (1) medication changes in the prior month; (2) significant current or recent (within the past year) CBSST or SST; (3) level of care at baseline that interferes with outpatient participation; (4) current hospitalization for psychiatric, substance use, or physical illness; (5) inability to pass a screening for cognitive impairment [18] during informed consent process; (6) inability to pass the 10-item T/F quiz about the details of the study informed consent, (7) current pregnancy; and (8) current incarceration.

Veterans will continue to receive ongoing care with their current providers, which for this population typically includes medication management, case management, and occasional psychotherapy targeting disorder-specific symptoms. This treatment will continue independent of the study, and no one involved in the study will make decisions regarding pharmacotherapy or other behavioral health treatments for the veteran. Participants will be withdrawn if they demonstrate a repeated pattern of non-participation and indicate they are no longer interested or do not respond.

### Randomization procedures and rationale

Once veterans are enrolled and have completed the baseline assessment, block randomization will be performed using a computer-generated list of numbers with a centralized web-based database system (Daciforms; Dacima Software Inc., Montreal, QC). RAs will use the database, set on the blinded status, to randomize enrolled participants once all prescreening information has been entered. Although veterans and the peer specialists will know to which condition the veterans have been assigned, RAs responsible for all assessments will be blinded to the study treatment assignment to prevent bias with subjective data collection measures in subsequent assessments. Data analysts will also be blinded.

### Measures

#### Patient outcomes

While the survey battery assesses symptoms, the measures mostly target functioning, recovery, and impacts that are applicable to both SST- and CBSST-Peer. We will use a multidimensional battery of functional outcomes: (1) self-report (Independent Living Skills Survey (ILSS)), (2) interviewer-rated (Abbreviated Quality of Life Scale (A-QLS); Brief Psychiatric Rating Scale (BPRS); Clinical Assessment Interview for Negative Symptoms (CAINS)), and (3) objective-indicator (Psychosocial Rehabilitation (PSR) Toolkit) measures of functioning that are impacted by both social and cognitive skills. See Table 1 for a complete list and description of patient outcome measures. We selected the ILSS (Table 2) as the primary outcome because it has been the primary outcome measure in prior CBSST studies that demonstrated the efficacy of CBSST in improving social and cognitive skills. We also focus on the patient experience of recovery because it is now viewed as critical and yet relatively independent of the traditional view of clinical change. Hence, studies of peer specialists as a recovery-promoting intervention must consider outcomes designed to capture a patient perspective, beyond those narrowly applied to traditional clinical domains (e.g., symptoms). In addition, we include a measure tied to both the SST- and CBSST-Peer interventions itself—the Comprehensive Modules Test (CMT)—to assess how well the veterans learn the social and cognitive content and can apply it to change their defeatist attitudes.

**Table 1:** Measures collected at baseline, 10 weeks, 20 weeks, and 32 weeks

Measure	Brief description
**Interview measures**	
**Abbreviated Quality of Life Scale (A-QLS)**	The A-QLS [19] is a 10-min semi-structured interview administered by a trained RA that measures subjective and objective aspects of functioning on 8 items in the past 4 weeks.
**Brief Psychiatric Rating Scale (BPRS)**	Assessed via clinical interview and subjectively rated by trained interviewers, the 24-item BPRS total score will be used to measure global psychopathology. The four BPRS positive symptom items—conceptual disorganization, suspiciousness, hallucinatory behavior, and unusual thought content—will be used to measure positive psychotic symptoms [20].
**Clinical Assessment Interview for Negative Symptoms (CAINS)**	Assessed via clinical interview and subjectively rated by trained interviewers, the 13 CAINS items are rated 0 (no impairment) to 4 (severe deficit) measuring the two negative symptom factors: expression and Motivation and Pleasure (MAP) across social, vocational, and recreational domains [21].
**Comprehensive Modules Test (CMT)**	The CMT is a 15-min interview assessing mastery of the content in the 2 CBSST modules and has been used in all prior CBSST trials. Questions with vignettes were developed to assess mastery of thought challenging (max = 11) and social communication (max = 11) skill knowledge. The CMT total score (max = 22) will be used.
**Psychosocial Rehabilitation (PSR) Toolkit**	The PSR Toolkit [22] is a 10-min interview used to collect information on employment, educational activity, and residential situation. In this brief interview, status in each functioning domain is rated on a progressive scale, ranging from the absence of meaningful functioning in the domain to fully independent functioning.
**Self-report measures**	
**Defeatist Performance Attitude Scale (DPAS)**	The DPAS is a 15-item, 5-min self-report subscale of the commonly used 40-item Dysfunctional Attitude Scale (DAS) derived from factor analysis. The DPAS indexes defeatist attitudes about one’s ability to perform tasks [23].
**Independent Living Skills Survey (ILSS) – primary outcome**	The ILSS [24] is a self-report measure of everyday functional living skills for patients with SMI that has proven to be reliable, stable, sensitive, and valid in multiple samples. The 51-item yes-no questionnaire takes less than 10 min to administer and assesses whether or not specific functioning behaviors have been performed over the past month.
**Patient Activation Measure (PAM)**	Patient activation is a self-report measure that refers to the knowledge, skills, confidence, and attitudes patients have for managing health and treatment. The shortened PAM is a 5-min,13-item measure in which respondents endorse items (e.g., “I know what each of my prescribed medications do”) on a scale from 1 (“disagree strongly”) to 4 (“agree strongly”) [25].
**Psychosocial Rehabilitation (PSR) Toolkit**	The PSR Toolkit [22] is a 10-min interview used to collect information on employment, educational activity, and residential situation. In this brief interview, status in each functioning domain is rated on a progressive scale, ranging from the absence of meaningful functioning in the domain to fully independent functioning.
**Recovery Assessment Scale (RAS)**	The RAS [26–28] is a 10-min checklist that assesses aspects of recovery with a special focus on hope and self-determination. The RAS has 41 items on which respondents rate themselves using a 5-point scale ranging from 1 = strongly disagree to 5 = strongly agree. The RAS is intended for use and has been tested with patients with SMI who receive services in outpatient settings and in peer-run programs.

**Table 2:** Study outcomes

Domain	Specific measurement	Specific metric	Method of aggregation	Time points
Everyday functioning	Independent Living Skills Survey (ILSS)	Difference in the change from baseline to each follow-up time point, between the three study groups	Mean	Baseline, 10 weeks, 20 weeks, 32 weeks

#### Fidelity

Two measures will be used to assess fidelity for CBSST-Peer and SST-Peer group sessions: Cognitive Therapy Rating Scale for Psychosis (CTS-Psy) [29] and the Social Skills Training Fidelity Scale (SSTFS) [30]. Both are observation checklists that have been used extensively to evaluate fidelity for CBSST and SST [11]. CBSST-Peer and SST-Peer sessions will be audio-taped and 20% rated for fidelity using these measures.

### Data collection, data entry, and data management

#### Study interview data

Trained RAs collect data during four assessments at weeks 0 (baseline), 10 (mid-intervention), 20 (end-of-intervention), and 32 (3-month post-intervention follow-up). All data will be maintained in the database using the internal data management tools. The database has been established in such a way that data entry errors (out of bound values) or fields accidentally left blank are immediately flagged. Veterans will be compensated up to $170 (baseline, $25; 10 weeks, $35; 20 weeks, $55; 32 weeks, $55) for completing the study visits.

Data will be kept behind the VA firewall or in locked file cabinets. Identified data will be shared only with regulatory agencies or for patient safety. De-identified data will be shared in compliance with the VA guidelines. Multiple attempts will be made to collect study data from enrolled participants. The study staff will monitor the participants for adverse events in multiple ways. Participants in the two intervention groups will be in close contact with peer specialists, who will report any adverse event they observe. Usual treatment participants will be observed during the four assessment visits and by contact with the participant’s assigned clinical providers.

#### Focus groups of veterans

Veterans in CBSST-Peer and SST-Peer conditions who indicated they would be willing to participate in a focus group at the time of consent will be invited to a face-to-face or virtual focus group once they have completed the study. Open-ended questions will ask about helpfulness and will focus on the utility of learning about errors in thinking and social skills. Participants of the CBSST-Peer group will be asked about whether there was any added impact of having the cognitive training over and above social skill training. There will also be questions asked about the benefit of having peer specialists run the groups (vs. non-peer providers). Saturation is generally reached with two groups of each “type” of participant [31]. To achieve saturation, four focus groups will be conducted (2 for SST-Peer; 2 for CBSST-Peer). Each group will last 60 min and include up to 8 participants, consistent with the established standards for successful focus groups [32]. Veterans will be randomly selected and consented for participation and audio taping and will be compensated $25 for their time. Each focus group will be audio-taped using a VA-approved recorder and transcribed verbatim.

#### Interviews of peer specialists and administrators

We will interview all participating peer specialists who delivered at least 10 sessions (anticipated to be *n* = 5) and all key mental health administrators (*n* = 4). These numbers will allow for saturation given they represent the full universe of peer specialists and key administrators from the study. These individuals will be asked about implementation barriers and facilitators. Specific probes will be drawn from the Consolidated Framework for Implementation Research (CFIR) [33]. The CFIR was chosen as it combines theories from across implementation science and covers five key domains: intervention characteristics, inner context (host organization), outer settings (environment-at-large), individuals involved, and the implementation process. Each domain is further divided into unique subdomains that provide areas to explore, as applied to SST- and CBSST-Peer—in other words, a defined list of specific barriers and facilitators shown to be important to implementation.

### Trial oversight

The study team will meet weekly to review the recruitment and retention progress. Our funder, the VA’s Health Services Research and Development (HSR&D) service, did not require additional trial oversight beyond what is already required, which is annual reporting to HSR&D and to the requisite IRB.

### Peer specialists

Peer specialists will be VA employees who are hired using the VA’s national guidelines for peer specialists, which specify the qualifying mental health conditions. These guidelines state that “a serious mental illness like schizophrenia, bipolar disorder, major depression, depression and anxiety disorders, post-traumatic stress disorder (PTSD), and substance use disorders qualify” [34]. Peer specialists will receive a full-day training in CBSST-Peer from CBSST experts. Part of the training involves developing their own examples to demonstrate the skills to be taught. For SST-Peer, each peer specialist must complete a 4-h online training course and pass the evaluation test. Then, an SST Master Trainer will hold a full-day training. After that, using audio tapes, the peer specialists will be evaluated and mentored over 6 months on their fidelity to the SST model. Peer specialists will receive weekly supervision on both interventions.

### CBSST-Peer condition

Peer specialists will co-lead a series of CBSST-Peer classes in two 10-class “modules”—the CBSST Cognitive Skills Module and the CBSST Social Skills Module—for a total of 20 weekly 60-min classes. These modules have been extensively tested and were used in the pilot. Veterans will continue to receive all other standard care, medication, and other services, regardless of group assignment, throughout the study. Standard CBSST integrates CBT and SST techniques and neurocognitive compensatory aids [35]. The treatment manual includes a patient workbook that describes the skills and includes homework assignments. Thought-challenging techniques are combined with role-play practice of communication skills. Compared to traditional CBT, much less training is required to become proficient at these thought-challenging skills. This is a standardized curriculum that can be delivered by most mental health providers, and the above adaptations make it especially appropriate for peer specialists. Cognitive interventions are used to address symptoms and challenge defeatist attitudes that interfere with real-world skills execution, including expectancies (“It won’t be fun”), self-efficacy attitudes (“I always fail”), and anomalous attitudes (“Spirits will harm me”) that interfere with skill performance, despite intact skill capacity. By challenging defeatist attitudes, patients are more likely to engage in functional behaviors and use the skills they have. To simplify learning and help patients remember and use cognitive techniques in everyday life, mnemonic aids are provided (e.g., laminated cards describing skills). For example, for thought challenging, we use an acronym, “The 3C’s: Catch it, Check it, Change it.” The “it” is a thought.

We have adapted standard CBSST to be delivered by peer specialists, capitalizing on the unique lived experiences of peer specialists to strengthen CBSST in several ways. First, peer specialists will provide personal examples of how they used the skills they are teaching to improve their own recovery. This makes the skills more tangible and less abstract, compensating for cognitive problems, and improving understanding of how specific skills can be used to achieve personal recovery goals. Peer specialists develop their examples as the part of the training, where they are given feedback about how to appropriately communicate the desired content. Second, engagement and retention of veterans with SMI in evidence-based psychosocial interventions is a persistent challenge and peer specialists can help overcome this barrier by instilling hope (“if they can use these skills to recover, I can too”). Hope and empowerment may improve motivation for treatment to overcome engagement and retention barriers. Third, more sessions have been added to allow for more practice time.

#### Recovery goal-setting session

Setting functional recovery goals is a key component of the CBSST program. During the first group session, veterans identify a long-term, personally meaningful recovery goal related to living, learning, working, or socializing. Together with the peer specialist, they divide their goal into short-term steps that can be accomplished each week. The skills training in the modules are then used to facilitate working on these steps. The goal-setting session is reviewed at the beginning of each module to review goal progress and develop new steps.

### Social skills training-peer condition

These groups will run according to the Bellack et al. model [30] used in VA’s national dissemination of SST. Peer specialists will receive the standard training that licensed independent practitioners (LIP) receive (peer specialists also are trained in SST, but current VA policy states they cannot deliver SST unless paired with an LIP). Similar to CBSST, these groups will use behavioral rehearsal role-play to improve communication and interaction skills. However, an important difference is that these groups will not include any cognitive training. There is flexibility in how long these groups run; however, to make the groups equivalent, we will make these groups last the same 20-week period.

### Analyses

#### Statistical analyses

Primary analyses will use hierarchical linear models (HLMs) for continuous variables. The HLMs will include random person-level intercepts and random time slopes. Possible random effects for cohort and peer specialist heterogeneity will be explored. Analyses will include all available data with maximum likelihood estimation, which is preferred over multiple imputations. All tests will be two-sided with alpha = 0.05.

#### Drop-out/retention

Based on our prior studies, we expect a dropout rate of 20% or less through the 32-week study period. Our prior trials with longer protocols had 86% retention after 18 months and 82% retention after 15 months. Retention will be aided by the provision of transportation. Assessments will be attempted with all participants, regardless of study retention. All analyses will follow an intention-to-treat approach that includes all participants randomized to a study group.

##### Aim 1: Effectiveness analysis and hypothesis

Analyses will test our hypothesis that veterans in CBSST-Peer will have superior functioning, recovery, attitude, and skill knowledge outcomes during treatment and follow-up relative to veterans in SST-Peer or TAU, and further that veterans in SST-Peer will have superior outcomes relative to TAU. Secondarily, we will apply all analyses to the symptom measures and explore the impact of gender and racial/ethnicity status where sample size allows. Analyses will follow the same pattern for all the above assessments. We will examine scores obtained at weeks 10 (mid-intervention), 20 (end-of-intervention), and 32 (post-intervention follow-up) as the outcome in HLM, with treatment condition included as a time-invariant covariate, and random intercepts for person and random slopes for time. All available data will be included in accordance with maximum-likelihood estimation procedures with data assumed missing-at-random, an assumption that will be tested with baseline clinical and demographic data. Preliminary analyses will examine potential baseline differences between the three conditions. Relevant covariates including treatment attendance, baseline symptom severity (via BPRS, CAINS), care services, and medications received (standard variables from the PSR toolkit), and demographic variables will be tested and included in the overall model according to model fit. The primary analysis will compare CBSST Peer to SST-Peer and TAU with independent contrasts in the HLM model of all post-baseline time points, to directly test the primary hypothesis that CBSST-Peer has superior outcomes than the other treatments (we will also compare SST-Peer to TAU). As compared to the group by time interaction, this test typically has greater power and is less susceptible to sample-specific artifacts (e.g., regression to the mean, variability in change over time). However, we also wish to examine the degree of change in outcomes over time in CBSST-Peer vs SST-Peer vs TAU. Thus, we will conduct additional analyses to test the treatment condition × time interaction, to examine the question of whether CBSST-Peer has greater improvement in outcomes over time than the other treatments.

##### Aim 1: Power

Power estimations are from the Stata power simulation package powersim. Data from previous trials and other SST trials were used to obtain effect size estimates to guide simulations of expected ILSS scores in CBSST-Peer, SST-Peer, and TAU. We first estimated power for statistically significant treatment main effect across various sample sizes, assuming 80% retention through the final 3-month follow-up assessment and *α* = 0.05 (two-tailed). For the treatment effects (CBSST-Peer vs. TAU; CBSST-Peer vs. SST-Peer; SST-Peer vs. TAU), we estimate small to medium effect sizes (*d* = 0.35) for ILSS over 20 weeks of treatment and 3-month follow-up. For *d* = 0.35 and retention of 80%, estimated power is 0.94 for *N* = 84 per treatment type and 0.83 for *N* = 67 per treatment type. This is consistent with previous trials of CBSST and SST in which CBSST produced the largest effect sizes (*d* = 0.649) and SST produced improvements, but lower effect sizes [36]. For each group × time interaction effect (comparing each group to each other over time), we project a medium (*d* =0.45) treatment type × time effect size, based on the assumption that CBSST-Peer will produce the largest increase in outcomes per month, based on our prior and other SST trials. For this effect size and our projected enrollment, power for the treatment type x time interaction is sufficient (> 0.80).

##### Aim 2: Data analysis

Each focus group and interview will be transcribed into verbatim text (removing identifying information like names or dates). Transcripts of the qualitative interviews and focus groups will be analyzed using rapid assessment, a team-based, iterative data collection and analysis approach [37]. A transcript summary template with domains based on the elements of the CFIR and other key topics will be collaboratively developed by the team. After testing and revising the template with at least the first five transcripts, a finalized version will be developed. Thereafter, we will independently use the summary template to closely read each transcript and summarize content under each domain, with periodic checks for consensus and reliability. The use of a template will facilitate the rapid but thorough review and reduction of data from several interviews, enabling the comparison and identification of themes both within and across study conditions, transcript type (focus group or interview) and stakeholder roles (peer specialist or key mental health staff). Furthermore, because preliminary analysis will begin as soon as the first transcripts are available, this approach will allow for iterative changes to the interview guides as new questions arise from the analysis. To establish that participants’ viewpoints and perceptions are accurately understood and reported, we will circulate the findings within our study team for further discussion and interpretation. Study findings will be disseminated via scientific journal articles, presentations at scientific conferences, and summaries for lay audiences circulated by our research center and will follow the authorship criteria of the International Committee of Medical Journal Editors.

## Discussion

Improving care and outcomes for individuals with SMI is a national priority and can be addressed using peer specialists delivering CBSST. Comparing peer specialist-delivered CBSST to peer specialist-delivered SST and usual care provides an opportunity to assess the limits of improvement in functioning. It also tests peer specialist’s unique ability to connect with individuals using a structured curriculum. This study addresses recommendations from SAMSHA that will both improve access and engagement in treatment. Lastly, this study preemptively addresses considerations needed to implement peer specialist-led CBSST on a larger scale. To our knowledge, this is the first effectiveness and implementation study assessing peer specialist services using CBSST or SST. If successful, this could improve care for individuals with SMI, change standards of care, and expand the breadth of services a peer specialist can provide.

## Trial status

This study is operating from protocol #10 with a recruitment start date of 2/18/2018 and end date of 12/31/2023. The trial is registered with ClinicalTrials.gov with an ID of NCT03467243.

## Supplementary Information

**Additional file 1.**

## Data Availability

Upon completion of the data collection, a final dataset with deidentified data will be made available as additional supporting files, in a machine-readable format.

## Abbreviations

A-QLS: Abbreviated Quality of Life Scale
BPRS: Brief Psychiatric Rating Scale
CAINS: Clinical Assessment Interview for Negative Symptoms
CBSST: Cognitive behavioral social skills training
CBT: Cognitive behavioral therapy
CDW: Corporate Data Warehouse
CFIR: Consolidated Framework for Implementation Research
CMT: Comprehensive Modules Test
CPRS: Computerized Patient Record System
CTS-Psy: Cognitive Therapy Rating Scale for Psychosis
DPAS: Defeatist Performance Attitude Scale
HLM: Hierarchical linear model
IRB: Institutional Review Board
ILSS: Independent Living Skills Survey
LIPs: Licensed independent practitioner
PAM: Patient activation measure
PS: Peer specialist
PSR: Psychosocial rehabilitation
PTSD: Post-traumatic stress disorder
RA: Research assistant
RAS: Recovery Assessment Scale
SAMHSA: Substance Abuse and Mental Health Services Administration
SMI: Serious mental illness
SST: Social skills training
SSTFS: Social Skills Training Fidelity Scale
TAU: Treatment as usual
VA: Veterans Affairs
