# Supplementary material for: Peer specialists deliver cognitive behavioral social skills training compared to social skills training and treatment as usual to veterans with serious mental illness: study protocol for a randomized controlled trial
Source: Trials. 2022 May 24;23:439. doi: 10.1186/s13063-022-06376-9 (PMC9128285; doi:10.1186/s13063-022-06376-9)
Supplement: Supplementary file 1 — Additional file 1. [file 13063_2022_6376_MOESM1_ESM.docx]

**Evaluating the Use of Peer Specialists to Deliver Cognitive Behavioral Social Skills Training**

Verbal (Phone) Consent - Veteran

Hello. My name is *[CIRB 17-47 team member]* and I am a team member with the study: *Evaluating the Use of Peer Specialists to Deliver Cognitive Behavioral Social Skills Training*. Is *[participant’s name]* there?

- **YES:** Continue below.
- **NO:** Leave a message with your name and number.

***OR*** *[if participant is returning a call]*

Hello, *[participant’s name]*. First, I want to thank you for your interest in the study. This call is to review what participating in the study involves and answer any questions. Afterwards, if you are still interested and willing to participate, we can continue with the verbal consent process.

You should have an information sheet about the study. Did you have a chance to review it?

- **YES:** Continue below.
- **NO:** Proceed only once the Veteran has possession of the information sheet/verbal consent form. Schedule a time to speak with the Veteran once they have these forms.
- **NO: Ask: would you like me to send another copy? Check the address is correct and ask If the Veteran could call when the materials arrive. Schedule another time to speak to the Veteran to check whether materials have arrived.**

After looking at the materials, are you still interested in finding out more about the study?

- **YES:** Continue below.
- **NO:** Thank you very much for your time. **STOP**

I’d like to go over some of the main parts of the study with you, and you can feel free to ask me any questions you have. As you saw on the study information sheet, your participation involves:

1. Being randomly assigned to one of three study groups: CBSST-Peer, SST-Peer, or Continued Behavioral Care as Usual

2. Having a total of 4 meetings over the course of 32 weeks with study staff where you will answer questions about your lives: how you are doing, your quality of life, recovery, and any mental health symptoms you may be experiencing. These visits will take about two hours and may be in-person or virtual.

3. If you are in the CBSST or SST groups, you will meet with a Peer Specialist for groups once a week for 20 weeks and will also agree to have these groups audio recorded. These recordings will be retained for data analysis. Additionally, if you are in the SST group, there is a chance that your recording may be used for training purposes.

4. If you are in the CBSST or SST groups and you provided permission, someone from the team may reach out to you about participating in a one-time, in-person or virtual focus group to talk about your experiences with CBSST-Peer and SST-Peer.

5. If you are assigned to Continued Behavioral Care as Usual, you will not participate in weekly group sessions, but will continue to receive the treatment you usually do for your serious mental illness.

Your participation is voluntary, and you can leave the study at any time. You are not required to answer questions from the surveys and interviews. If you leave the study, any collected data will be kept by the study team. We will write about the combined data we have gathered and will not individually identify any Veterans.

The information collected for this study will be kept confidential. Appropriate measures will be taken to ensure that your identity remains confidential. There are times when we might have to show your records to others, for example, from the Office of Human Research Protections, the Government Accountability Office, the Office of the Inspector General, the VA Office of Research Oversight, the VA Central IRB, our local Research and Development Committee, and other study monitors may look at or copy portions of records that identify you.

You may not directly benefit from participating. You may receive group treatment at no cost. You may feel better and function better than with standard medical treatment alone. Your participation may also help the VA learn how well the treatments in this study work for Veterans.

There are no expected direct benefits from participating in the interviews or focus groups. There is a potential benefit of finding out how helpful CBSST-Peer and SST-Peer are for Veterans. The VA may benefit by learning how to put these programs into practice.

You will be paid for your time and participation in the study. You will be paid for four research visits, but you will not be paid for participating in groups.

Now I would like to ask you a few questions before we continue: *Study staff to administer the BOMC or Blessed Evaluation as well as the Consent Verification document prior to obtaining verbal consent.*

*Veteran passed screening_______ Veteran did not pass screening_______*

*Veterans who fail the screening will be thanked, paid, and told they are not eligible for the study.*

Do you have any questions or concerns about the study?

- **YES:** Answer questions.
- **NO:** Continue below.

Do you consent to participate in this study?

- **YES:** Thank you for agreeing to enroll in this study*.* Research staff will be in contact with you about next steps. Thank you for agreeing to be a part of the study. We will now put a note in your medical record saying that you have agreed to participate in this study.
- **NO:** Unfortunately, we cannot enroll anyone in this study without obtaining their informed consent. Thank you for your interest in the study. Please feel free to contact us at any time if you would like to be a part of the study in the future or if you have any further questions. **STOP**

If you are in the CBSST or SST groups, are you willing to be contacted about participating in a future virtual focus group:

____Yes, I am willing to be contacted about participating in the focus group

____No, I do not want to be contacted about participating in the focus group

PARTICIPANT CONSENTED TO STUDY: **YES NO** SITE: ______________________

NAME: ___________________________________ LAST 4 SSN: ____ ____ ____ ____

CONSENT OBTAINED BY: ______________________________ ________________________

Study Staff Signature Print

DATE CONSENT OBTAINED: ____ ____ / ____ ____ / ____ ____ ____ ____
